# Replicon family of *Vibrionaceae* plasmids as a reservoir of antimicrobial and phage resistance genes in marine ecosystems

**DOI:** 10.1093/ismejo/wraf274

**Published:** 2025-12-10

**Authors:** Soraya Fraga-Pampín, Carlos R Osorio, Ana Vences

**Affiliations:** Aquatic One Health Research Center, and Department of Microbiology and Parasitology, Universidade de Santiago de Compostela, Santiago de Compostela 15782, Spain; Aquatic One Health Research Center, and Department of Microbiology and Parasitology, Universidade de Santiago de Compostela, Santiago de Compostela 15782, Spain; Aquatic One Health Research Center, and Department of Microbiology and Parasitology, Universidade de Santiago de Compostela, Santiago de Compostela 15782, Spain

**Keywords:** plasmid replication, *Vibrionaceae*, antimicrobial resistance, replicon typing, phage defense systems, marine microbiology

## Abstract

Plasmids are mobile genetic elements that drive horizontal gene transfer among bacteria, influencing microbial community composition and functional traits in marine ecosystems. However, many marine plasmids remain unclassified due to unknown replication mechanisms. Here, we describe VBR1, a novel plasmid replicon family, widespread among species of the family *Vibrionaceae*. The minimal VBR1 replicon comprises a 570-bp AT-rich origin of replication (*oriV*) and two genes, *vrp1AB*, sufficient for autonomous replication in *Escherichia coli* and *Photobacterium damselae*. A comprehensive GenBank search revealed 158 previously untyped plasmids from *Vibrionaceae* species worldwide harboring this replicon, including relevant pathogens for animals and humans as well as environmental species. VBR1 plasmids share a syntenic set of backbone genes, are predominantly conjugative, and frequently encode antimicrobial resistance (AMR) genes, conferring resistance to multiple antibiotic classes. Most VBR1 plasmids also carry phage defense and anti-defense systems, underscoring their ecological and evolutionary significance. AMR and defense/anti-defense gene repertoires are highly variable across VBR1 plasmids, suggesting frequent gene acquisition, recombination events, and rapid replacement and diversification of resistance and defense determinants. The co-localization of AMR and phage defense systems on many VBR1 plasmids highlights their role in shaping virus–host interactions and microbial community dynamics. Our findings establish VBR1 as a widespread, clinically and ecologically relevant replicon family, providing a framework for the classification and surveillance of previously orphan plasmids, and advancing our understanding of AMR and phage resistance dynamics in marine ecosystems.

## Graphical Abstract

**Graphical Abstract:**
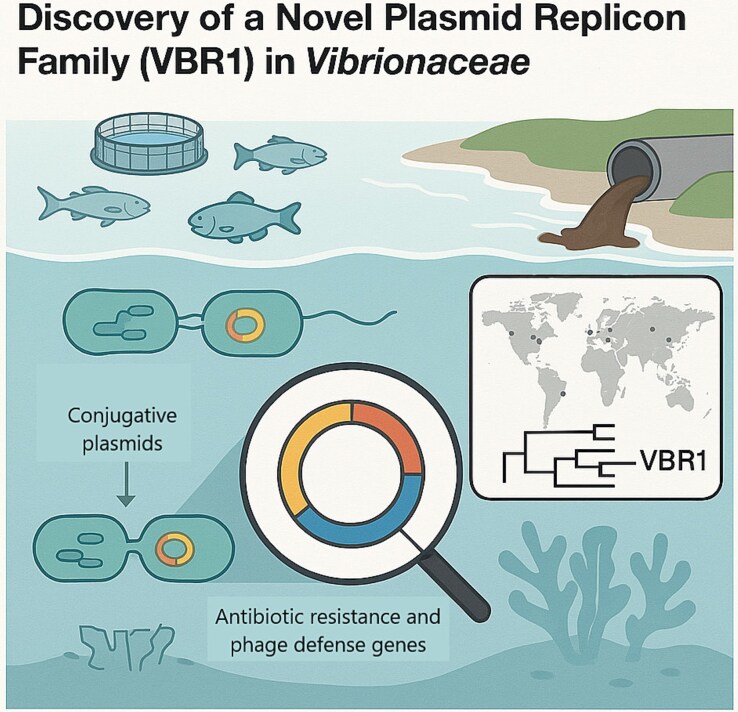

## Introduction

The family *Vibrionaceae* (commonly referred to as “vibrios”) comprises more than 190 species of Gram-negative rods, primarily inhabiting marine and estuarine ecosystems [1]. Vibrios play important roles in aquatic environments, contributing to nutrient cycling through the degradation of organic matter such as chitin and engaging in diverse interactions with marine organisms, ranging from mutualistic symbioses to pathogenic associations. Their capacity to rapidly respond to fluctuations in temperature, salinity, and nutrient availability makes them valuable indicators of environmental change [2, 3]. Although most vibrios are non-pathogenic, a subset includes species pathogenic to humans, and others can infect marine animals, thereby impacting aquaculture of fish, mollusks, and crustaceans [2]. Global ocean warming has been linked to an increase in infectious diseases caused by vibrios in both humans and marine organisms [3]. Marine ecosystems receive runoff from agricultural activities and wastewater effluents, serving as reservoirs of microbial diversity and resistance genes [4, 5]. Additionally, the use of antibiotics in aquaculture contributes to the spread of antimicrobial resistance (AMR) [6]. In vibrios, this represents a growing concern, with resistance genes located on mobile genetic elements, including Integrating Conjugative Elements [7, 8], plasmids [9, 10], and Class 1 integrons [11, 12].

The evolutionary arms race between prokaryotes, bacteriophages, and mobile genetic elements has driven the diversification of microbial defense and counter-defense strategies [13], and over 150 defense systems (and a similar number of anti-defense systems) have been identified in prokaryotes and their phages [14, 15]. Vibrios experience intense phage predation, and rapid turnover of mobile, chromosome-borne genomic islands encoding defense systems can dramatically alter phage susceptibility in *Vibrio* populations [16, 17]. However, the role of plasmids as reservoirs and vectors of these systems remains poorly understood, particularly in marine microbial communities. Marine plasmids remain scarcely studied and are underrepresented in public databases, because research on plasmid replication mechanisms and incompatibility group classification has historically focused on *Enterobacteriaceae* and other clinically relevant bacteria [18].

Characterizing the replication origin (*oriV*) of plasmids is essential for understanding their copy number, host range, incompatibility, persistence, and dissemination [19], and for developing plasmid interference strategies to combat AMR [20]. Some plasmids of vibrios have been assigned to IncP, IncQ, and IncC incompatibility groups [21–23]. A plasmid family, MRB (Marine RNA-based), is restricted to *Vibrionaceae* species [24]. However, classification into replicon families remains unaccomplished for most *Vibrio* plasmids.

In this study, we identified the minimal replicon (designated VBR1) shared by the virulence plasmid pPHDP70 of *Photobacterium damselae* subsp. *piscicida* and the multidrug resistance plasmid pAQU1 of *P. damselae* subsp. *damselae*. Extensive database searches revealed 158 GenBank entries with VBR1 plasmids across *Vibrionaceae* species. Most encode multiple antibiotic resistance genes and diverse phage defense and anti-defense systems. This finding enables classification of previously untyped *Vibrionaceae* plasmids from environmental and pathogenic strains. We propose that VBR1 plasmids contribute to the evolutionary success of various *Vibrionaceae* lineages by providing a broad repertoire of anti-phage defense systems and AMR mechanisms.

## Materials and methods

### Bacterial strains and growth conditions

Strains used in this study are described in Supplementary Table S1. *Escherichia coli* strains were grown on tryptic soy agar (TSA) or broth (TSB) at 37°C. *P. damselae* subsp*. damselae* (*Pdd*) and subsp*. piscicida* (*Pdp*) strains were grown at 25°C on TSA and TSB supplemented with 0.5% NaCl (TSA-1 and TSB-1, respectively). Antibiotics were used at the following concentrations: kanamycin (Kn) at 50 *μ*g/ml, ampicillin (Ap) at 100 *μ*g/ml, tetracycline (Te) at 12 *μ*g/ml, chloramphenicol (Cm) at 20 *μ*g/ml, rifampicin (Rf) at 25 *μ*g/ml, nalidixic acid (Nl) at 40 *μ*g/ml.

### PCR, plasmid construction, and identification of the VBR1 minimal replicon

Plasmids and primers are described in Supplementary Tables S1 and S2, respectively. Polymerase chain reaction (PCR) amplifications were performed using the NZY*Taq* II 2× Green Master kit (NZYTech). For colony PCR, an isolated colony was suspended in 50 *μ*l H_2_O and 3 *μ*l added to the PCR tube. Three candidate nucleotide regions from pPHDP70, designated *seq1*, *seq2*, and *seq3*, were PCR-amplified and cloned into pWKS30 or ligated to a kanamycin resistance cassette (Kn^R^) obtained from pKD4. Constructs were introduced into *E. coli* DH5*α* via heat-shock transformation [25], and into *Pdp* DS11 by electroporation [26]. Plasmid DNA was prepared using the NZYMiniprep kit (NZYTech). PCR products were purified using the NZY Gelpure kit (NZYTech). Autonomous replication of genetic constructs was assessed by growth of transformant cells in Kn^50^, followed by plasmid recovery, PCR tests for sequence integrity, and plasmid stability in the absence of selection. Chemically synthesized potential mini-replicons (GenScript Biotech Corporation, Piscataway, NJ, USA) containing either *vrp1A + vrp1B + oriV*, *vrp1B* + *oriV*, and *vrp1A* + *oriV* were ligated to Kn^R^ gene and tested for self-replicative ability. *vrp1A* and *vrp1B* are the two ORFs encoded within *seq1*, whereas *seq3* has been renamed as *oriV* (see below).

### Conjugation assays

Bacterial donor and recipient strains were grown for 24 h with the appropriate antibiotics. One milliliter of cultures of donor strains and 2 ml of recipient strains adjusted to an OD600 of 1.0 were centrifuged, cells mixed in 100 *μ*l TSB-1, and the mixture dispensed onto a TSA plate prepared with seawater (TSA-SW). Cells were mated for 1 day at 37°C for *E. coli* strains, and at 25°C when strains of *P. damselae* were involved. Serial dilutions were plated on TSA-1 plates with the corresponding antibiotics to count donors, recipients, and transconjugants. Transfer frequencies were calculated as the number of transconjugants per donor cell. Conjugation experiments were carried out in triplicate, and data correspond to the mean value of three biological replicates.

### Plasmid compatibility assays

Compatibility between VBR1 plasmids was tested in a conjugation assay using *Pdd* containing pPHDDOG2 (Te^R^) as donor, and a rifampicin resistant *Pdp* strain (DI-21Rf^R^) carrying pPHDP70 as recipient. A multiplex PCR targeting the pPHDP70 *frpA* gene [27] and the pPHDDOG2 *tetB* gene [10] was conducted for the screening of 200 Rf^R^ Te^R^ transconjugants. Compatibility of VBR1 and IncC plasmids was assessed by mating *E. coli* XL1-Blue containing IncC plasmid pKAZ3 with *E. coli* CCW012 (MC1061 containing pPHDP70::Kn^R^) [28] and by mating *Pdd* OG15A containing VBR1 plasmid pPHDDOG15A, with *E. coli* CAG18420 (pKAZ3).

### Plasmid stability assays

Stability assays were performed using pPHDDOG2 (Te^R^, Cm^R^) and pPHDDOG15A (Cm^R^) [10] VBR1 plasmid as model. The *Pdd* native host strains (OG2, OG15A) were serially subcultured four times in antibiotic-free TSB-1 (~20 generations per subcultivation cycle), and then cultures were plated on TSA-1. Three hundred colonies per strain were randomly picked on TSA-1 containing Cm to assess plasmid presence.

### Database mining and sequence analysis

BLASTn and tBLASTn searches were performed using *vrp1AB* genes and the 570-bp *oriV* as independent queries against the GenBank nucleotide database (last updated 1 July 2025). Plasmid annotations were manually curated, and backbone synteny was assessed using pAQU1 as a reference. Comparative genomic analyses were performed using Easyfig v.2.2.3. [29]. MOTIF, InterproScan, and Alphafold databases were used for prediction of domains in replication proteins Vrp1A and Vrp1B.

### Prediction of antibiotic resistance determinants and defense systems

Antibiotic resistance genes were predicted using the Comprehensive Antibiotic Resistance Database (CARD) [30]. Defense and anti-defense systems were identified using DefenseFinder, AntiDefenseFinder tools [14, 15], and PADLOC [31]. Heatmaps of gene presence/absence were constructed using Chiplot [32].

### Phylogenetic analyses

Evolutionary analyses were performed within MEGA11 (v 11.0.13) [33]. Amino acid sequences of Vrp1AB homologs were obtained using National Center for Biotechnology Information (NCBI) Basic Local Alignment Search Tool (protein) (BLASTp). Distantly related RepA sequences from IncC plasmid pKAZ3 and IncA plasmid pRA1 were added to Vrp1AB datasets as outgroup. Nucleotide sequences of the 570-bp *oriV* of VBR1 plasmids were obtained using NCBI Basic Local Alignment Search Tool (nucleotide) BLASTn. For phylogenetic tree construction, sequences were aligned with MUSCLE [34]. For amino acid sequences, we used the Maximum Likelihood method (PhyML) [35] and the Le and Gascuel matrix-based model [36] with gamma distribution (LG + G). Initial trees for heuristic search were obtained automatically by applying Neighbor-Join and BioNJ algorithms to a matrix of pairwise distances estimated using a Jones-Taylor-Thornton (JTT) model, then selecting the topology with superior log likelihood value. Percent identity values were retrieved from multiple sequence comparison by log-expectation (MUSCLE) output [34]. For *oriV* (nucleotide sequence), evolutionary history was inferred using Neighbor-Joining method. Evolutionary distances computed using Jukes-Cantor method are in units of number of base substitutions per site.

### Environmental surveillance of VBR1 replicons

Primers targeted to conserved sequences of *vrp1AB* and *oriV* were designed (Supplementary Table S2) and tested using genomic DNA of *P. damselae* strains harboring VBR1 plasmids. Samples of European sea bass (*Dicentrarchus labrax*) gills and gut content, seawater, and biofilm from seabass cage nets were obtained from sea bass aquaculture facilities in the Spanish Mediterranean coast. DNA was extracted from gills and intestinal content using the NZY tissue gDNA isolation kit (NZYTech). Seawater and biofilm samples were cultured in TSB-1 for 24 h at 25°C, and a dilution was used directly as template. PCR reactions were conducted using the NZY*Taq* II 2× Green Master kit (NZYTech), supplemented with 1% (vol/vol) dimethyl sulfoxide (DMSO), programming 30 cycles of: denaturation at 95°C (60 s), annealing at 56°C (30 s), and elongation at 72°C (20 s).

## Results and discussion

### Identification of a marine plasmid replicon type in *P. damselae*

Multidrug-resistant strains of the fish and human pathogen *P. damselae* subsp. *damselae* have been isolated from aquatic environments in Asia and the Black Sea, with resistance determinants harbored on a family of homologous conjugative plasmids, typified by pAQU1 [10, 12, 37, 38]. pAQU1 shares conserved backbone genes with IncA/C plasmids, including genes for conjugative transfer, DNA repair, regulation, entry and surface exclusion, an observation that led to its tentative classification as untyped IncA/C-like plasmid [39]. However, the sequences governing replication of typical IncA/C plasmids are not found in pAQU1.

**Figure 1:**
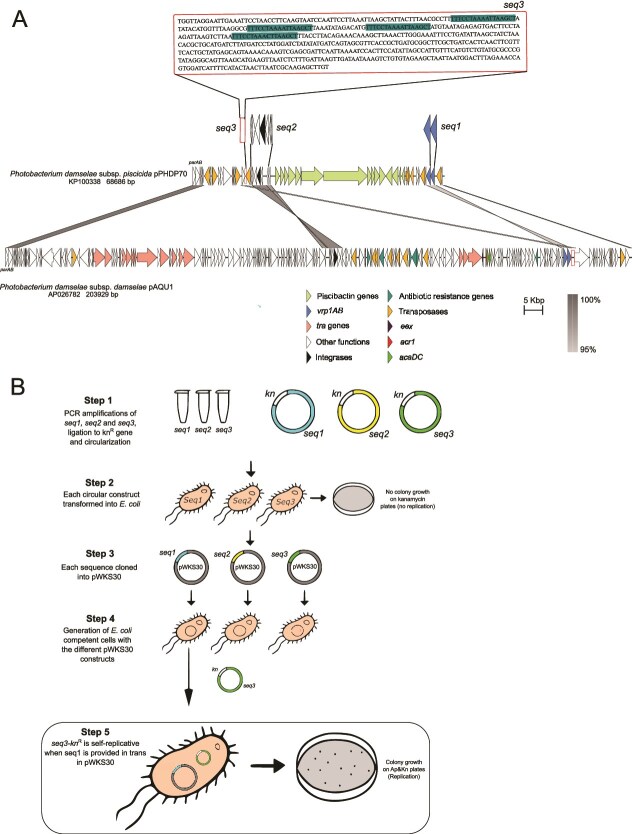
Characterization of a novel plasmid replicon family (VBR1) in *Vibrionaceae* species; (A) comparative analysis of plasmids pPHDP70 and pAQU1, showing the conserved gene modules; the minimal replicon required and sufficient for replication, as demonstrated in this study, consists of: (1) *seq1*, including *vrp1A* and *vrp1B*, and (2) *seq3*, the *cis*-acting origin of replication (*oriV*) (shown as a red-bordered open rectangle); the nucleotide sequence of *seq3* is expanded, and the four direct repeats are highlighted; (B) cloning strategies to identify the VBR1 minimal replicon.

pAQU1 shares four sequence modules with pPHDP70, a virulence plasmid encoding siderophore piscibactin in the fish pathogen *P. damselae* subsp. *piscicida* [28]. These modules comprise 14 genes with 98% nucleotide identity, encoding hypothetical proteins, and partitioning functions (Fig. 1A). By conjugative assays, we were unable to obtain transconjugants that stably maintained pPHDP70, and the pAQU-like plasmid pPHDDOG2 (Table 1), suggesting that they share replication functions. Given its smaller size relative to pAQU1, pPHDP70 was selected to investigate the minimal replication requirements, under the hypothesis that replication relies on conserved sequences shared by both plasmids. Three pPHDP70 regions were evaluated as *cis*-acting origin of replication or *trans*-acting elements: (i) *seq1*, a 2.8-kb region encoding two hypothetical proteins (GenBank WP_044179386.1 and GenBank WP_069107784.1) designated Vrp1A and Vrp1B (Vrp stands for *Vibrio*  replication protein), respectively; (ii) *seq2*, a 6.8-kb region encoding an integrase and five hypothetical proteins; and (iii) *seq3*, a 570-bp adenine and thymine (AT)-rich region (dubbed *oriV*) flanked by *IS* elements, and containing four direct repeats (Fig. 1A).

**Table 1:** Results of conjugative transfer experiments using VBR1 plasmids and an IncC plasmid, in different combinations of donor and recipient bacterial strains and species.

Donor strain name	Donor strain description	Recipient strain name	Recipient strain description	Transfer frequency
*Photobacterium damselae* subsp*. damselae* OG2	VBR1 plasmid pPHDDOG2; Te^R^, Cm^R^	*Photobacterium damselae* subsp. *piscicida* DI-21Rf^R^	Contains VBR1 plasmid pPHDP70	3.2 × 10^−3^^a^
*Escherichia coli* XL1-Blue pKAZ3	Contains IncC plasmid pKAZ3; Te^R^, Ap^R^	CCW012 (*Escherichia coli* MC1061 + pPHDP70::Kn)	Contains VBR1 plasmid pPHDP70; Kn^R^	1.1 × 10^−5^
*Escherichia coli* XL1-Blue pKAZ3	Contains IncC plasmid pKAZ3; Te^R^, Ap^R^	*Escherichia coli* MC1061	Plain *Escherichia coli* with no plasmids	1.8 × 10^−5^
*Photobacterium damselae* subsp*. damselae* OG15A	VBR1 plasmid pPHDDOG15A; Cm^R^	*Escherichia coli* CAG18420	Kn^R^	1.9 × 10^−4^
*Photobacterium damselae* subsp*. damselae* OG15A	VBR1 plasmid pPHDDOG15A; Cm^R^	*Escherichia coli* CAG18420 + pKAZ3	Contains IncC plasmid pKAZ3; Kn^R^, Te^R^, Ap^R^	ND
SFP_pOG15A	*Escherichia coli* CAG18420 transconjugant for pPHDDOG15A; Te^R^, Cm^R^	*Escherichia coli* MG1655	Nl^R^	1.6 × 10^−4^

a All transconjugants showed to retain pPHDDOG2 but lost pPHDP70. Although *Pdp* DI-21Rf^R^ that acquired pPHDDOG2 were obtained at a frequency of 3.2 × 10^−3^ transconjugants per donor cell, a screening of 200 Rf^R^ Te^R^ transconjugants using a multiplex PCR targeting markers of pPHDP70 (*frpA* gene) and of pPHDDOG2 (*tetB* gene) revealed the presence of pPHDDOG2 and the absence of pPHDP70 in 100% of the colonies, indicative of pPHDP70 curing and of the incompatibility between the two plasmids.

We found that *seq3* ligated to Kn^R^, was self-replicative in *E. coli* cells on condition that cells were previously transformed with pWKS30 carrying *seq1* (Fig. 1B). To further demonstrate that the three elements (*oriV*, *vrp1A*, and *vrp1B*) are necessary for replication, distinct combinations were chemically synthesized and tested for self-replicative ability. We found that *vrp1A + vrp1B* + *oriV +* Kn^R^ was the only construction that yielded Kn^R^ colonies of *E. coli* and of *P. damselae* subsp. *piscicida* DS11 upon transformation, providing evidence that the three elements are necessary in both *E. coli* and in *Vibrionaceae* genetic backgrounds. When we electroporated *P. damselae* subsp. *piscicida* DS11 with a 1:1 mixture of *vrp1A + oriV +* Kn^R^, and *vrp1B* + *oriV +* Kn^R^, all the Kn^R^ colonies proved to contain both constructs simultaneously. These results provided strong evidence that *vrp1AB* encode the *trans*-acting elements, whereas *seq3* functions as the *cis*-acting *oriV* origin of replication. Vrp1A and Vrp1B did not show similarity to known Rep proteins from characterized replicon types. A search for conserved domains only retrieved an AAA-family ATPase domain (PF13481) (AAA stands for ATPases Associated with diverse cellular Activities) for Vrp1B.

In pPHDP70, a plasmid that has undergone a massive expansion of *IS* elements, the minimal replicon is split into two separate regions, whereas in pAQU1 and related plasmids, the replicon constitutes a contiguous sequence (Fig. 1A). The *cis*-acting *oriV* sequences of pPHDP70 and pAQU1 showed 94% nucleotide identity. The amplification product of the minimal replicon region from the pAQU-like plasmid pPHDDOG2 [10], ligated to Kn^R^, proved to be self-replicative in *E. coli* and in *P. damselae* subsp. *piscicida* DS11 when introduced by electroporation (data not shown). We designated this novel replicon type as VBR1 (*Vibrio-*based replicon 1). The VBR1 replicon sequence from pPHDDOG2 has been deposited in GenBank under accession number PV872854, with curated annotations of functional elements. This reference enables the unambiguous identification of VBR1-related plasmids through similarity searches, allowing researchers to retrospectively classify previously untypeable plasmids and to recognize VBR1 affiliation in newly sequenced elements.

### VBR1 replicon highly conserved in antimicrobial resistance plasmids of *Vibrionaceae* species globally

The GenBank database mining analysis identified 158 entries with >94% identity to *vrp1AB* and *oriV* sequences, all corresponding to species of *Vibrio* and *Photobacterium* (*Vibrionaceae* family) (Table 2). No homologous sequences were found in *Enterobacteriaceae*, *Aeromonadaceae*, or other bacterial families. The VBR1 replicon was detected in 22 species distributed across diverse regions worldwide (Table 2). These findings suggest a specific association between the VBR1 replicon and *Vibrionaceae* species.

**Table 2:** Summary of 158 *Vibrio* and *Photobacterium* entries containing VBR1-type replicons.

Organism/plasmid name	Size (bp)	Source, country, year	Acc. number	Refs.
**DATASET 1**				
*Photobacterium damselae* subsp*. piscicida*, pPHDP70	68 686	Gilthead seabream, Spain,1990	KP100338.1	[28]
*Photobacterium damselae* subsp*. damselae,* pAQU1	203 929	Sea water, Kagawa (Japan), 2004	AP026782.1	[37]
*Vibrio* sp*. 04Ya090,* pAQU2	160 406	Sediment, Japan, 2004	AB856327.1	[38]
*Vibrio parahaemolyticus*, pVPH1	183 730	Shrimp, Hong Kong, 2010	KP688397.1	[40]
*Vibrio parahaemolyticus*, pVPH2	198 487	Shrimp, Hong Kong, 2011	KP791968.1	[12]
*Vibrio parahaemolyticus*, pVPS43	194 479	Shrimp, Shenzhen, 2013	KX957970.1	[12]
*Vibrio parahaemolyticus*, pVPS91	163 005	Shrimp, China, 2015	KX957972.1	[12]
*Vibrio parahaemolyticus*, pVPS62	184 719	Chicken, Shenzhen, 2015	KX957971.1	[12]
*Vibrio alginolyticus*, pVAS114	206 274	Shrimp, Shenzhen, 2015	KX957969.1	[12]
*Vibrio alginolyticus,* pVAS19	187 130	Shrimp, Shenzhen, 2015	KX957968.1	[12]
*Vibrio penaeicida*, pTUMSATOK2	150 127	Kuruma shrimp, Okinawa, 2019	AP025157.1	[41]
*Vibrio penaeicida,* pTUMSATOK1	150 136	Kuruma shrimp, Okinawa, 2019	AP025154.1	[41]
*Vibrio harveyi,* p1	153 407	*Lates calcarifer*, Chennai, 2020	CP125877.1	[42]
*Vibrio parahaemolyticus*, pVPSD2016-2	193 123	*Litopenaeus vannamei*, Weifang, 2016	CP034301.1	[43]
*Vibrio owensii,* p2	186 279	Shrimp, 2016	CP030801.1	[44]
*Vibrio alginolyticus,* pC704	193 433	Shrimp, China	OP958859.1	[45]
*Vibrio alginolyticus,* pUnnamed1	217 123	Seawater, Puerto Rico	CP014052.1	[45]
*Vibrio harveyi,* p345-185	185 327	*Epinephelus *lanceolatus**, Shenzhen, 2013	CP025539.1	[46]
*Vibrio parahaemolyticus*, pVb677-tet	183 307	Shrimp, China, 2015	OQ622008.1	[47]
*Vibrio alginolyticus*, pC1579	236 774	Shrimp, Shenzhen, 2016	MN865127.1	[44]
*Vibrio parahaemolyticus*, pLH80-1	83 353	*Litopenaeus vannamei*, Tangshan, 2017	CP068629.1	[48]
*Vibrio furnissii*, pMT14	207 270	*Ruditapes philippinarum,* China, 2021	CP115190.1	[49]
*Vibrio* sp*. YMD68,* pUnnamed1	168 227	Sediment, Yellow Sea, 2022	CP124615.1	
*Vibrio parahaemolyticus,* p_2743	156 346	Shrimp, India, 2017	CP066163.1	
*Vibrio parahaemolyticus*, p1 = pVp94-1	191 858	Maricultured shellfish, China, 2023	CP080480.1	[50]
*Vibrio alginolyticus,* pUnnamed1	270 467	Coastal soil, South Korea, 2024	CP167181.1	
*Vibrio parahaemolyticus*, pVP7-1	229 659	Seafood, Nanjing, 2021	CP150866.1	
*Vibrio alfacsensis*, plas2	221 646	*Scophthalmus maximus*, Weihai, 2021	CP140105.1	[51]
*Vibrio parahaemolyticus*, pA-vp-201 806	157 684	*Litopenaeus vannamei*, Fujian, 2018	CP150859.1	
*Vibrio alginolyticus,* pUnnamed1	217 099	Mollusc, Canada, 2015	LFWL01000057.1	
*Vibrio harveyi,* pVh21-1	203 647	*Paralichthys olivaceus*, South Korea, 2021	JAOWIO010000003.1	
*Photobacterium damselae* subsp. *damselae*, pPHDDOG15A	183 310	*Dicentrarchus labrax*, Black Sea, 2011	VANG00000000.1	[10]
*Photobacterium leiognathi*, pSr2.36_1	166 153	*Siphamia roseigaster*, Australia, 2023	CP183260.1	
*Photobacterium leiognathi,* pSr2.4_1	166 167	*Siphamia roseigaster*, Australia, 2023	CP183275.1	
*Vibrio parahaemolyticus*, pUnnamed	158 668	*Penaeus vannamei*, Ecuador, 2021	CP176033.1	[52]
*Vibrio cyclitrophicus,* pUnnamed2	182 882	Filtered seawater, Massachusetts, 2010	CP170592.1	[53]
*Vibrio cyclitrophicus,* pUnnamed2	182 884	Marine, Massachusetts, 2010	CP170040.1	[54]
**DATASET 2**				
*Vibrio vulnificus* Per5 9	155 245	Water, Albufera (Spain), 2023	NZ_JBBEOE010000009.1	[55]
*Vibrio vulnificus* Permar1 9	155 245	Water, Albufera (Spain), 2023	NZ_JBBEOA010000009.1	[55]
*Vibrio alginolyticus* BII-4F	123 723	*Saccostrea cucullata*, Mauritius, 2017	NZ_JACEOF010000017.1; NZ_JACEOF010000018.1	[56]
*Vibrio parahaemolyticus* MAVP-21	173 067	*Homo sapiens*, 2013	NIXV01000011.1	[57]
*Vibrio diabolicus* HS-50-2	157 134	Oyster, Canada, 2016	NZ_JAMQQV010000001.1	[58]
*Vibrio parahaemolyticus* F6_1 F6_1	116 751	Water, Guangxi, 2014	NZ_NNEP01000032.1	[59]
*Vibrio mediterranei* 21LN0615E	157 094	Water, France, 2015	NZ_NWTO01000014.1	[60]
*Vibrio vulnificus* VA-WGS-18060	135 744	Estuarine water, Texas (USA), 2007	NZ_RBWN01000012.1	[61]
*Vibrio antiquarius* 939	141 161	Oyster, Washington (USA), 2007	NZ_AOJB01000001.1	[62]
*Vibrio cholerae* HS-119-4	172 271	Clams, Quebec (Canada), 2018	NZ_JAMPYI010000050.1	[58]
*Vibrio* sp. 1078-1	162 638	Food, China, 2016	NZ_JAWOWM010000011.1	
*Vibrio* sp. 665	162 638	Food, China, 2015	NZ_JAWOUI010000022.1	
*Vibrio albus* E4404	241 083	Marine sediments, Weihai, 2016	NZ_QFWT01000007.1	[63]
*Vibrio* sp. 1942	165 514	Food, China, 2016	NZ_JAWOVG010000013.1	
*Vibrio* sp. Vb2135	165 634	Food, China, 2017	NZ_JAWOSS010000012.1	
*Vibrio* sp. 1408	162 539	Food, China, 2016	NZ_JAWPOU010000019.1; NZ_JAWPOU010000016.1	
*Vibrio* sp. 1557	159 884	Food, China, 2016	NZ_JAWOVZ010000010.1	
*Vibrio* sp. 2089	165 523	Food, China, 2016	NZ_JAWOVA010000012.1	
*Vibrio* sp. 661	165 522	Food, China, 2015	NZ_JAWOUJ010000010.1	
*Vibrio* sp. 704	165 666	Food, China, 2015	NZ_JAWOUH010000020.1; NZ_JAWOUH010000021.1	
*Vibrio* sp. Vb0301	161 272	Food, China, 2015	NZ_JAWOTU010000006.1	
*Vibrio* sp. YT-19	167 896	Food, China, 2015	NZ_JAWOQN010000010.1	
*Vibrio* sp. 1974	142 178	Food, China, 2016	NZ_JAWPOQ010000032.1; NZ_JAWPOQ010000027.1	
*Vibrio mytili* CAIM 528	167 299	Seawater, Spain, 1985	NZ_JXOK01000015.1	[64]
*Vibrio* sp. Vb2736	139 425	Food, Shenzhen, 2018	NZ_JAFLNP010000014.1	
*Vibrio alginolyticus* Vb2145	164 328	Food, Shenzhen, 2017	NZ_JAFLNX010000011.1	
*Vibrio parahaemolyticus* Vb0849	168 154	Food, Shenzhen, 2016	NZ_JAFLOD010000011.1	
*Vibrio parahaemolyticus* Vb0825	168 154	Food, Shenzhen, 2016	NZ_JAFLOE010000012.1	
*Vibrio parahaemolyticus* Vb1382	161 560	Food, Shenzhen, 2016	NZ_JAFLNY010000011.1	
*Vibrio parahaemolyticus* Vb1081	153 557	Food, China, 2016	JARKGT010000008.1	[65]
*Vibrio parahaemolyticus* GCSL_R8	156 215	Oyster, Texas (USA), 2007	MIQL01000005.1	[66]
*Vibrio cholerae*	166 179	USA	AAXNBW010000005.1	[67]
*Vibrio parahaemolyticus*	193 884	Crab meat lump, Venezuela, 2018	AAXNZJ010000008.1	[68]
*Vibrio parahaemolyticus*	167 004	Oyster, Washington (USA), 2013	AAXODT010000003.1	[68]
*Vibrio parahaemolyticus*	167 007	Oyster, Washington (USA), 2012	AAXOPO020000012.1	[68]
*Vibrio parahaemolyticus*	150 631	*Crassostrea gigas*, UK, 2010	AAXOXP020000008.1	[68]
*Vibrio parahaemolyticus*	151 642	USA, 2000	AAXPAS020000013.1	[68]
*Vibrio parahaemolyticus*	168 891	USA	ABACHX010000010.1	[67]
*Vibrio parahaemolyticus* MA303	158 956	Oyster, Massachusetts (USA), 2015	DACQMO010000015.1	[69]
*Vibrio parahaemolyticus* VP12075	155 026	*Homo sapiens*, Shenzhen, 2012	DAHTME010000012.1	[69]
*Vibrio parahaemolyticus* VP04098	153 774	*Homo sapiens*, Shenzhen, 2004	DAHUJG010000014.1	[69]
*Vibrio cholerae* M183306	150 444	*Homo sapiens*, Australia, 2018	DAMYAO010000005.1	[69]
*Vibrio parahaemolyticus* BT79-19	154 876	*Litopenaeus vannamei*, Vietnam, 2020	NZ_JASKZO010000015.1	[70]
*Vibrio parahaemolyticus* BT76-45	154 876	*Litopenaeus vannamei*, Vietnam, 2020	NZ_JASKZP010000014.1	[70]
*Vibrio parahaemolyticus* Vb957	182 444	Food, Shenzhen, 2016	JARKCB010000010.1	[47]
*Vibrio parahaemolyticus* Vb2111	165 523	Food, China, 2017	JARKEO010000012.1	[47]
*Vibrio vulnificus* IRLA0161	172 360	Sediment samples, Florida, 2019	NZ_JAERHX010000012.1	[71]
*Vibrio parahaemolyticus* C6_9 C6_9	168 110	Fish, Shandong, 2014	NZ_NNKK01000033.1; NZ_NNKK01000025.1	[59]
*Vibrio parahaemolyticus* F4_10	133 137	Sediment samples, Guangxi, 2014	NZ_NNFH01000089.1; NZ_NNFH01000071.1; NZ_NNFH01000009.1	[59]
*Vibrio anguillarum* 00-84-1	157 247	*Panopea generosa*, Washington, 2000	NZ_VTYO01000025.1; NZ_VTYO01000019.1	[72]
*Vibrio splendidus* 1F_55	162 937	Seawater, Massachusetts, 2006	NZ_PIGA01000010.1	
*Vibrio parahaemolyticus* E4_2 E4_2	141 475	Fish, Zhejiang, 2014	NZ_NNHG01000007.1; NZ_NNHG01000006.1; NZ_NNHG01000005.1	[59]
*Vibrio parahaemolyticus* VP365	172 902	*Homo sapiens*, Zhejiang, 2013	NZ_JACBKG010000015.1; NZ_JACBKG010000012.1	[73]
*Vibrio parahaemolyticus* A11	159 691	*Litopenaeus vannamei*, Guangdong, 2022	NZ_JAPXUY010000022.1; NZ_JAPXUY010000021.1	[74]
*Vibrio* sp. Vb0877	146 887	Food, Shenzhen, 2016	NZ_JAFLOC010000031.1; NZ_JAFLOC010000024.1	
*Vibrio sinensis* BEI233	146 887	Eastern China Marginal Sea, 2017	NZ_QVMU01000030.1; NZ_QVMU01000024.1	[75]
*Vibrio* sp. 1401	139 925	Food, China, 2016	NZ_JAWOWF010000044.1; NZ_JAWOWF010000016.1	
*Vibrio* sp. 705	170 480	Food, China, 2015	NZ_JAWOUG010000017.1; NZ_JAWOUG010000013.1	
*Vibrio* sp. Vb1166	196 964	Food, China, 2016	NZ_JAWOTE010000019.1; NZ_JAWOTE010000017.1	
*Vibrio parahaemolyticus* Vb1207	167 821	Food, Shenzhen, 2016	NZ_JAFLNZ010000021.1; NZ_JAFLNZ010000018.1	
*Vibrio parahaemolyticus* PNUSAV001588	145 292	Oyster, USA, 2021	ABAYSP010000030.1; ABAYSP010000020.1	[67]
*Vibrio parahaemolyticus* PNUSAV001391	154 157	Shrimp, Missouri (USA), 2021	ABDZHN010000012.1	[76]
*Vibrio parahaemolyticus* V21010073	146 795	Oyster, New Jersey (USA), 2021	ABEVGL010000010.1	[68]
*Vibrio parahaemolyticus* V21010069	146 785	Oyster, New Jersey (USA), 2021	ABEVGX010000021.1; ABEVGX010000013.1	[68]
*Vibrio parahaemolyticus*	149 231	Oyster, New Jersey (USA), 2023	ABLZJR010000013.1	[67]
*Vibrio harveyi* va43845-2018	153 288	*Homo sapiens*, Germany, 2018	DANSCA010000034.1; DANSCA010000018.1	[69]
*Vibrio parahaemolyticus* V189	177 070	Squid, Hongkong, 2016	JABCCI010000015.1; JABCCI010000013.1	
*Vibrio parahaemolyticus* Vb801	166 283	Food, China, 2015	NZ_JARKCV010000025.1; NZ_JARKCV010000019.1; NZ_JARKCV010000014.1	[47]
*Vibrio parahaemolyticus* Vb1004	171 048	Food, China, 2016	JARQQC010000009.1	[47]
*Vibrio* sp. 945	180 938	Food, China, 2015	JAWOTY010000013.1; JAWOTY010000003.1	
*Vibrio cholerae* V130039	172 346	Japan, 2013	DAILRS010000007.1	[69]
*Vibrio metschnikovii*	184 312	Shrimp, Missouri (USA), 2020	ABKWPU010000028.1; ABKWPU010000016.1; ABKWPU010000014.1	[76]
*Vibrio fluvialis* VF096	171 886	Environment, Anhui, 2018	JAHUEK010000010.1	[77]
*Vibrio harveyi* 9567-98	181 908		JACGMG010000006.1	[78]
*Vibrio parahaemolyticus* VP375	162 575	Stool sample, Shenzhen, 2010	JADQIJ010000012.1	[79]
*Vibrio parahaemolyticus* Vb0826	168 154	Food, China, 2016	JARKAD010000014.1	[80]
*Vibrio parahaemolyticus* MA267	184 600	Environment, Massachusetts, 2015	DACQJM010000004.1	[69]
**DATASET 3**				
*Vibrio parahaemolyticus* F4_7 F4_7	49 459	Water, Guangxi, 2014	NZ_NNFB01000463.1; NZ_NNFB01000153.1; NZ_NNFB01000142.1; NZ_NNFB01000141.1	[59]
*Vibrio parahaemolyticus* 10 290	79 808	*Homo sapiens*, China, 2010	NZ_CAJNZM010000088.1; NZ_CAJNZM010000071.1; NZ_CAJNZM010000070.1; NZ_CAJNZM010000058.1	
Uncultured *Vibrio* sp. 611	9524	Marine macroalgae, Weihai, 2018	NZ_CANLMY010000146.1	
*Vibrio parahaemolyticus* vp-CTS7-1	106 170	Shrimp, Shenzhen, 2023	NZ_JBBFBE010000121.1; NZ_JBBFBE010000118.1	
*Vibrio parahaemolyticus* A7	159 691	*Litopenaeus vannamei*, Guangdong, 2022	NZ_JAPXUU010000022.1; NZ_JAPXUU010000021.1	[81]
*Vibrio parahaemolyticus* A8	159 691	*Litopenaeus vannamei*, Guangdong, 2022	NZ_JAPXUV010000020.1; NZ_JAPXUV010000019.1	[81]
*Vibrio* sp. 1457	75 572	Food, China, 2016	NZ_JAWOWA010000023.1	
*Vibrio parahaemolyticus* N2-5	169 168	*Oratosquilla oratoria*, Shanghai, 2022	NZ_JALGSI010000024.1; NZ_JALGSI010000021.1	
*Vibrio* sp. SG41-7	62 406	Estuary, Chesapeake Bay (USA), 2018	NZ_JACJFF010000177.1; NZ_JACJFF010000150.1; NZ_JACJFF010000135.1; NZ_JACJFF010000121.1; NZ_JACJFF010000108.1	
*Vibrio* sp. 1456-1	73 690	Food, China, 2016	NZ_JAWOWB010000023.1	
*Vibrio* sp. 708	94 398	Food, China, 2015	NZ_JAWOUE010000017.1	
*Vibrio* sp. 736	94 398	Food, China, 2015	NZ_JAWOUD010000014.1	
*Vibrio* sp. 707	94 398	Food, China, 2015	NZ_JAWOUF010000015.1	
*Vibrio parahaemolyticus* F6_9 F6_9	78 525	Fish, Guangxi, 2014	NZ_NNEH01000108.1; NZ_NNEH01000075.1; NZ_NNEH01000058.1	[59]
*Vibrio* sp. 1579	51 060	Food, China, 2016	NZ_JAWOVU010000037.1	
*Vibrio anguillarum* FXH-305	58 873	Seawater layers, Wuhan, 2021	NZ_JAJGNT010000023.1	
*Vibrio parahaemolyticus* SD2016121	112 332	*Homo sapiens*, Haici hospital, 2016	NZ_JAEPRU010000020.1; NZ_JAEPRU010000014.1	
*Vibrio parahaemolyticus* K1314	144 366	USA, 2004	NZ_LHBE01000104.1; NZ_LHBE01000087.1	
*Vibrio vulnificus* vv-83	107 084	Shrimp, Beijing, 2016	DACQFZ010000038.1; DACQFZ010000034.1; DACQFZ010000022.1	[69]
*Vibrio parahaemolyticus* ISF-331-1	112 776	Raw shrimp, Bangladesh, 2019	NZ_JANFRQ010000176.1; NZ_JANFRQ010000150.1; NZ_JANFRQ010000038.1	
*Vibrio parahaemolyticus* 210083	144 220	Oyster, New Jersey (USA), 2017	AAZQSG010000003.1	[68]
*Vibrio parahaemolyticus* V22053613	52 399	Oyster, New Jersey (USA), 2022	ABLZKI010000039.1	[68]
*Vibrio parahaemolyticus* Vb677	118 725	Food, China, 2015	JARQRQ010000016.1	[47]
*Vibrio parahaemolyticus* 582-14	142 417	*Homo sapiens*, Perú , 2014	JAGJKA010000047.1; JAGJKA010000026.1; JAGJKA010000019.1	
*Vibrio vulnificus* CFSA-31	124 113	Fish, Beihai, 2018	DAPTMC010000015.1	[69]
*Vibrio cholerae*	162 749	USA, 2021	ABBYXK010000007.1	[67]
*Vibrio parahaemolyticus* SH15-1	152 382	Mallard, Guangdong, 2018	NZ_JAQBJA010000009.1	[82]
*Vibrio parahaemolyticus* SH15-2	152 382	Mallard, Guangdong, 2018	NZ_JAQBJB010000012.1	[82]
*Vibrio parahaemolyticus* SH16-1	152 382	Mallard, Guangdong, 2018	NZ_JAQBJC010000009.1	[82]
*Vibrio parahaemolyticus* SH16-2	152 382	Mallard, Guangdong, 2018	NZ_JAQBJD010000009.1	[82]
*Vibrio parahaemolyticus* 33-1 33-1	152 366	*Charadriiformes*, Guangdong, 2019	NZ_JAQBLH010000014.1	[82]
*Vibrio parahaemolyticus* 33-2 33-2	152 366	*Charadriiformes*, Guangdong, 2019	NZ_JAQBLI010000013.1	[82]
*Vibrio parahaemolyticus* 26-2 26-2	152 097	Mallard, Guangdong, 2019	NZ_JAQBKW010000013.1	[82]
*Vibrio parahaemolyticus* VPS2 12	152 779	*Litopenaeus vannamei*, Thailand, 2017	NZ_JASSJY010000012.1	
*Vibrio parahaemolyticus* F7_8 F7_8	145 385	Fish, Guangxi, 2014	NZ_NNEA01000021.1	[59]
*Vibrio parahaemolyticus* E1_5 E1_5	155 206	Fish, Zhejiang, 2014	NZ_NNHR01000030.1	[59]
*Vibrio alginolyticus* Va145	131 453	Seawater, China, 2007	NZ_JANJMF010000026.1; NZ_JANJMF010000020.1	
*Vibrio alginolyticus* Vb1165	84 153	Food, Shenzhen, 2016	JAFLOA010000019.1	
*Vibrio vulnificus* Z-31	24 448	Fish, Beihai, 2018	JAPWRK010000031.1	
*Vibrio parahaemolyticus* Vb0986	169 398	Food, China, 2016	JARQQD010000008.1	
*Vibrio sp.* ECSMB14106	54 824	Marine biofilm, Zhoushan, 2012	LAUM01000016.1	
*Vibrio campbellii* VB18PR-0122-1	55 294		DANSCJ010000037.1	[69]
*Vibrio splendidus* FF-6 FF-6	134 832	Seawater, Massachusetts, 2006	NZ_AJZI02000041.1; NZ_AJZI02000202.1	
*Vibrio alginolyticus* 2020RZ141	80 949	Ocean, 2020	NZ_JBBLMX010000023.1	

The table combines three datasets: **Dataset 1**: 37 closed plasmid sequences annotated as such in GenBank and identified in this study as members of the VBR1 group. **Dataset 2**: 77 GenBank entries not annotated as plasmids but identified as containing a VBR1-type replicon. In 23 cases, contigs were manually assembled into putative plasmids using pAQU1 as a reference. **Dataset 3**: 44 GenBank entries containing VBR1-type replicons for which complete plasmid assemblies could not be proposed due to missing backbone elements. These are considered partial or fragmented plasmid sequences.

Among the 158 entries, 37 represent complete sequences annotated as plasmids from *Photobacterium* and *Vibrio* strains originating from diverse geographical locations and isolation sources including marine animals, seawater, and sediments (dataset 1 in Table 2). Of these, 30 plasmids have been described in published studies, although their replicon types were often ambiguously defined. Some were referred to as pAQU-like plasmids, whereas others lacked any reference to plasmid classification. This inconsistency has hindered the systematic assignment of relevant AMR plasmids to specific families. Based on our findings, *Vibrio harveyi* p345–185, *Vibrio parahaemolyticus* pVb677-tet [46, 47], and Plas2 from *Vibrio alfacsensis*, a turbot pathogen isolated from aquaculture effluents [51], can be confidently classified within the VBR1 group. The same applies to five plasmids of *Vibrio alginolyticus* and *V. parahaemolyticus* that were previously reported to carry AMR genes embedded within complex Class 1 integrons [12]. Similarly, the recently described *Vibrio furnissii* plasmid pMT14, which harbors the *bla*  _GMA-1_ gene encoding a mobile Class A *β*-lactamase (GMA-1) [49], and plasmid pC1579 from a foodborne *V. alginolyticus* isolate encoding the novel metallo-*β*-lactamase VAM-1 [44], are assignable to the VBR1 group. The latter study highlighted the inability to classify pC1579 using PlasmidFinder [18] and emphasized its backbone similarity to IncC plasmids. These examples underscore the utility of VBR1 *oriV* identification as a robust tool for classifying previously untyped AMR plasmids.

A second dataset (Table 2) comprises 77 GenBank entries not annotated as plasmids. In most cases, the associated publications (when available) did not mention the presence of plasmids. However, 23 sequences appeared to represent incomplete plasmid assemblies. We manually examined the contig collections linked to these 23 entries, and using pAQU1 as a reference, reconstructed putative plasmid architectures based on backbone gene synteny (contigs including VBR1 sequences are listed in Table 2). These newly identified VBR1 plasmids include elements from human and animal pathogens as well as from environmental non-pathogenic vibrios, including the recently described species *Vibrio sinensis* and *Vibrio albus*. This dataset expands the known geographical distribution of VBR1 plasmids to previously unreported regions, including Spain, France, Germany, the UK, Mauritius, Vietnam, the USA, Canada, and Venezuela. The dataset also provides evidence of VBR1 plasmids in a *Vibrio mytili* isolated in 1985 in the Atlantic coast of Spain and in a *Vibrio mediterranei* from 2015 to 2016 in the Mediterranean Sea [60]. Furthermore, our study reveals the presence of VBR1 plasmids in two isolates of the zoonotic pathogen *Vibrio vulnificus* from the Albufera lagoon in Valencia, Spain.

The human pathogen *V. parahaemolyticus* is prominently represented in this second dataset. Although recent studies have documented AMR in clinical isolates, the associated plasmids have often remained untyped. This includes three plasmids from the serotype O4:KUT2, recovered from patients with diarrhea [73], all of which can be assigned to the VBR1 group. Additionally, the Foodborne *V. parahaemolyticus* Genome Database (FVPGD), a repository dedicated to the genomic surveillance of this pathogen in China, is poised to become a valuable resource for risk assessment and outbreak investigation [83]. Based on our findings, we recommend curating the FVPGD to incorporate the VBR1 plasmids identified in the present study. Moreover, the potential for global dissemination of this plasmid group is highlighted by the detection of VBR1 plasmids in *V. parahaemolyticus* recovered from fecal samples of migratory birds [84].

A third dataset includes 44 GenBank entries that lack the backbone elements found in the plasmids listed in dataset 2 and likely are fragments of VBR1 plasmids that remain incomplete due to assembly gaps. Many of these entries are associated with studies describing antibiotic-resistant vibrios isolated from fish and crustacean aquaculture facilities, human clinical samples, ducks, seagulls, and seafood. In addition to the major human pathogens, this dataset includes plasmids from *Vibrio campbellii*, *Vibrio splendidus*, and *Vibrio anguillarum*.

### VBR1 family includes conjugative plasmids encoding multidrug resistance determinants

The sizes of the 114 plasmids listed in datasets 1 and 2 range from 70 to 270 kb (Supplementary Fig. S1). The smallest include the non-conjugative virulence plasmid pPHDP70 and plasmid pLH80-1, which shares a backbone with pAQU1 but lacks the variable region encoding antibiotic resistance genes (Table 2). This size range is broader than the 160–206 kb reported in a previous work based on only nine representatives [12], underscoring the diversity captured in the expanded dataset of the present study.

**Figure 2:**
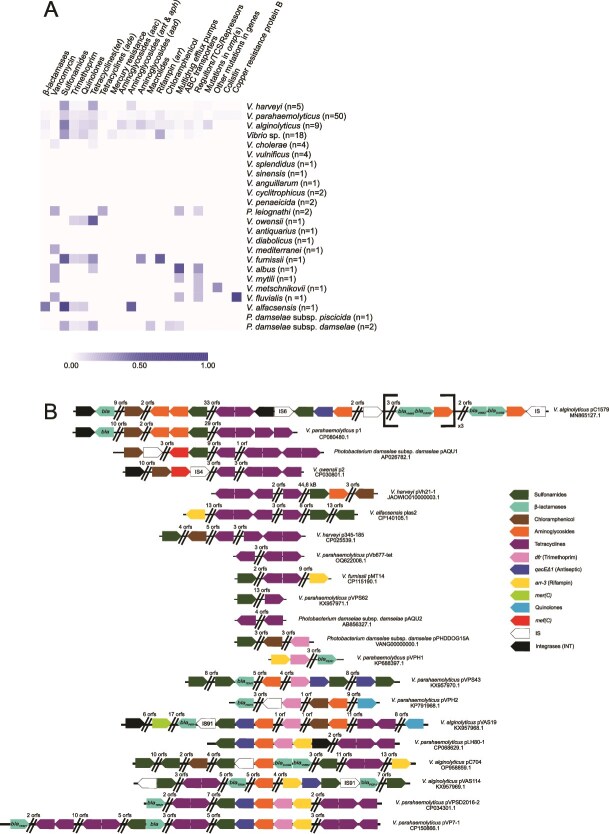
Analysis of antibiotic resistance genes in VBR1 plasmids; (A) heatmap showing the presence/absence profiles of antibiotic resistance genes across 114 *Vibrionaceae* strains; antibiotics are grouped by class for clarity, and strains are organized by species; shades of blue indicate the frequency of resistance genes within each class, calculated as the proportion of positive strains per species; (B) genetic organization of antimicrobial resistance clusters in 21 fully assembled VBR1 plasmids (see Table 2).

Except for pPHDP70, all VBR1 plasmids encode a set of *tra* genes, being potentially conjugative as has been experimentally demonstrated for some of them [10, 37]. The majority of the 114 VBR1 plasmids in datasets 1 and 2 carry genes conferring resistance to multiple antibiotic classes, including aminoglycosides, macrolides, tetracyclines, quinolones, sulfonamides, *β*-lactams, phenicols, thrimethoprim, and quaternary ammonium compounds, as predicted by the CARD database (Fig. 2A), with the genes *sul2*, *tetR*, *qnrVC6*, *qnrS2*, *tetB*, and *tetM* being particularly abundant (Supplementary Fig. S2). Recent work identified the *qnrS2* gene in plasmid pC704 as a distinctive marker, previously thought to be absent in related plasmids [45]. Similarly, *qnrVC*, first detected within an integron in *Vibrionaceae* in 2008 was found in a substantial number of VBR1 plasmids. *qnrS* and *qnrVC* genes are supposed to be derived from *Vibrio* spp. ancestors and have been found in diverse bacterial taxa, both in chromosomal and mobile elements locations [84].

Comparative analysis of the organization of AMR gene clusters in 21 representative VBR1 plasmids revealed that each plasmid carries a distinct combination of resistance genes (Fig. 2B), indicative of frequent gene acquisition, recombination events, and rapid turnover of resistance determinants. Collectively, these findings highlight the diversity of resistance genes harbored by VBR1 plasmids, many of which had previously remained unclassified. Climate change and the widespread use of antibiotics are contributing to the rise of AMR in vibrios [3], consistent with the global distribution of strains harboring VBR1 plasmids (Table 2). This trend is also evident in aquaculture systems worldwide, where fish, crustaceans, and shellfish farming environments serve as reservoirs of resistance genes [85].

Due to the fragmented nature of the assemblies, it was not feasible to reliably detect AMR genes within the unassembled contigs of most VBR1 plasmids in dataset 3. For this reason, the heatmap (Fig. 2A) includes only plasmids from datasets 1 and 2. This limitation stems from the frequent association of resistance genes with repetitive DNA elements, which hinder contig resolution in short-read sequencing projects.

The conjugative transfer of VBR1 plasmids among *Vibrio* populations may contribute to the emergence of strains resistant to clinically important antibiotics used to treat *Vibrio* infections [9, 86], as well as to antimicrobials commonly employed in aquaculture [87]. Consequently, monitoring the dissemination of VBR1 plasmids is strongly recommended. Such surveillance can be achieved with high specificity by targeting conserved sequences within the VBR1 minimal replicon using custom-designed oligonucleotides. In this study, two sets of VBR1-specific primers were validated with pure bacterial cultures and field samples, enabling the detection of this replicon family in fish specimens and in the environment of a sea bass aquaculture facility (Supplementary Fig. S3). This methodological framework is particularly timely, as VBR1 plasmids were primarily reported in Asian aquatic environments until recently [10, 12]. Our current findings demonstrate their recent detection in geographically distant regions, suggesting a rapid and ongoing global dissemination. Except for two cases, all available VBR1 plasmid sequences are associated with biosamples collected after the year 2000. An illustrative example is provided by *Vibrio cholerae* isolates harboring VBR1 plasmids, all sampled between 2013 and 2021. Despite the availability of more than 14 000 *V. cholerae* genomes from historical collections spanning several decades, these plasmids have not been detected in older isolates. This absence suggests that the emergence and geographic spread of this plasmid family within *Vibrio* may be a relatively recent event. This emerging trend underscores the need for proactive monitoring strategies to trace the spread of VBR1 plasmids.

### VBR1 plasmids encode diverse phage defense and anti-defense systems

**Figure 3:**
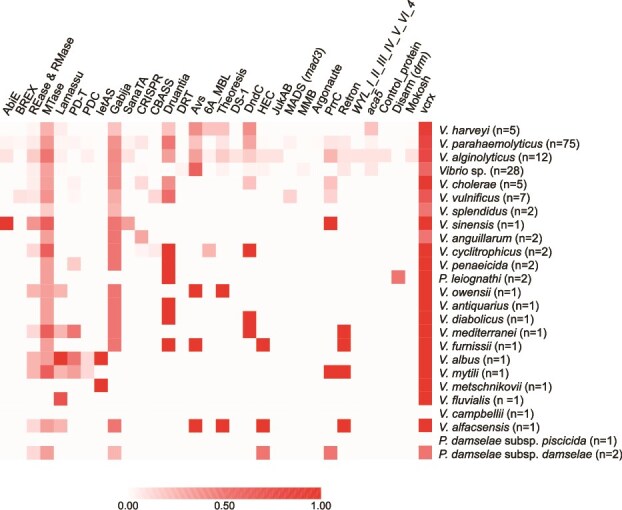
Heatmap illustrating the presence/absence profiles of defense and anti-defense systems across 158 *Vibrionaceae* strains; systems are grouped by class for clarity, and strains are organized by species; shades of red indicate the occurrence frequency of defense or anti-defense genes within each class, calculated as the proportion of positive strains per species.

We analyzed VBR1 plasmids using PADLOC as well as the DefenseFinder and AntiDefenseFinder tools, revealing that many plasmids encode both types of systems (Fig. 3; Supplementary Fig. S4). Among the defense mechanisms, we identified strategies based on (a) foreign nucleic acid targeting, including restriction-modification (RM), clustered regularly interspaced palindromic repeats (CRISPR)-Cas, and Gabija systems [88–90]; and (b) abortive infection (Abi) systems [91], which trigger growth arrest or cell death upon foreign DNA invasion, thereby suppressing replication and limiting the spread of the invading element. These include, among others, the AbiEii, CBASS, Thoeris, pAgo_GbbAgaS, SIR2, ThsA, Avs, and Lamassu elements detected in VBR1 plasmids. A previous study reported two untyped *V. parahaemolyticus* plasmids encoding a type IV CRISPR-Cas system [92], which we now classify as members of the VBR1 group (Table 2). However, research on antiphage defense in *Vibrionaceae* has focused primarily on chromosomal systems, such as integrons in *V. parahaemolyticus* [93] and *V. cholerae* [94], and genomic islands in *Vibrio lentus* and *V. cholerae* [16, 95–97]. Thus, our study represents a breakthrough in the previously underestimated role of plasmids as carriers of defense systems against foreign DNA in *Vibrionaceae*. The abundance and diversity of antiphage systems capable of spreading via conjugative plasmids may shape the evolutionary arms race between *Vibrio* populations and their phage predators, and could significantly challenge the development of phage therapy strategies against vibrios [16].

From the perspective of a bacterial cell acting as a recipient in conjugative mating, plasmids can be perceived as invasive elements (foreign DNA) comparable to bacteriophages. Consequently, plasmids have developed anti-defense mechanisms that enable them to bypass bacterial immune systems [98]. Five different families of anti-defense mechanisms have been predicted among VBR1 family (Fig. 3; Supplementary Fig. S4). In total, 82% (130 out of 158) of VBR1 plasmids harbor the anti-defense modules *vcrx089*-*vcrx090* and *vcrx092*-*vcrx093*, a percentage that might increase pending the completion of plasmid assemblies in dataset 3. This high prevalence suggests that these systems play a pivotal role in the interactions between VBR1 plasmids and their bacterial hosts. These genes were recently characterized in IncC plasmids [99]. Genes *vcrx089* and *vcrx090* confer resistance to type I RM systems, whereas *vcrx092*-*vcrx093* (encoding a single-strand annealing recombinase and a double-strand exonuclease, respectively) constitute a CRISPR-Cas evasion system. The absence of *vcrx091* (encoding a single-strand binding protein) in all the VBR1 plasmids suggests that it is not essential for double-strand DNA breaks repair, as previously reported [99]. Presence of these anti-defense mechanisms likely broadens the host range of VBR1 plasmids, allowing them to evade CRISPR-Cas immunity and RM systems encoded in potential recipient cells.

### VBR1 family includes plasmids with coherent phylogenies based on replicative genes

**Figure 4:**
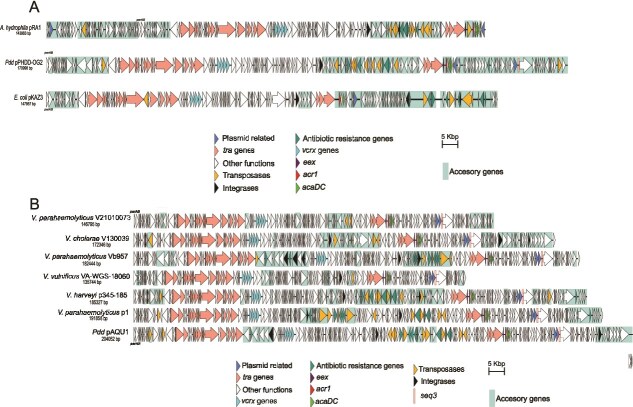
Comparative genomic analysis of VBR1 plasmids; (A) comparison of the VBR1 plasmid pPHDDOG2 with IncA (pRA1) and IncC (pKAZ3) plasmids; (B) alignment of seven representative VBR1 plasmids; in both panels A and B, backbone genes, including those involved in conjugation (*tra*), DNA repair (anti-defense *vcr*x** genes), and entry/surface exclusion systems (*eex*, *acr1*, *acaDC*), are represented with different color codes. Accesory genes are shaded in blue.

**Figure 5:**
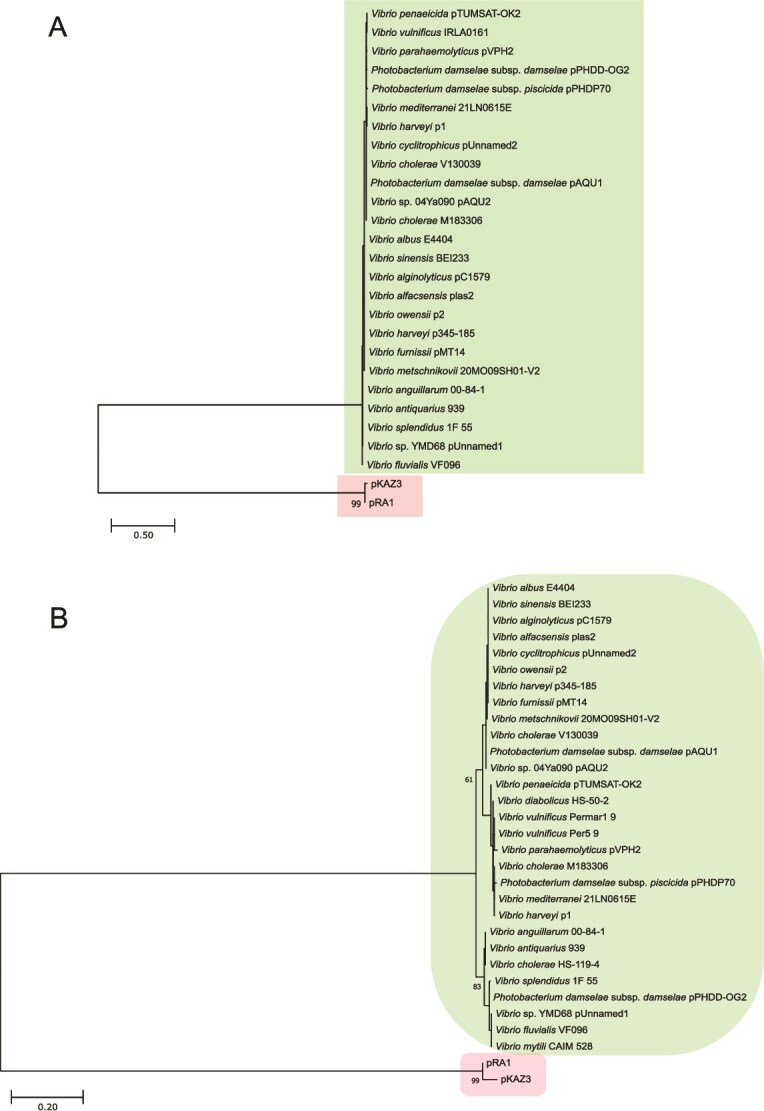
Phylogenetic analysis of Vrp1A (A) and Vrp1B (B) proteins from VBR1 plasmids. Distantly related RepA sequences from IncC plasmid pKAZ3 and IncA plasmid pRA1 were added to Vrp1AB datasets as outgroup.

A recent study proposed the classification of pAQU1 as an untyped relative of IncA and IncC plasmids based on backbone similarities [39]. In our analysis, the majority of the 158 VBR1 plasmids shared with typical IncA and IncC members a syntenic set of backbone genes involved in conjugative transfer, DNA repair, regulation, entry and surface exclusion (Fig. 4A). However, each plasmid also contains variable cargo DNA encoding adaptive traits, including AMR genes when present (Fig. 4B). Phylogenetic trees of Vrp1A (Fig. 5A) and Vrp1B (Fig. 5B) sequences revealed clusters of closely related VBR1 plasmids, clearly separated from RepA sequences of IncA and IncC representatives providing strong evidence for the definition of VBR1 as a novel replicon type. When IncA/C RepA proteins were not included as outgroup, the phylogenetic trees of Vrp1AB revealed incipient trends of sequence divergence among VBR1 plasmids. Similar levels of sequence divergence were found when comparing the non-coding *oriV* sequences (Supplementary Fig. S5).

The lack of homology between the replicons of IncA/IncC and VBR1 plasmids suggests they may belong to distinct incompatibility groups. However, incompatibility is a broad term that encompasses replication mechanisms, exclusion (entry and surface), and partition systems. These concepts are often conflated in the literature, leading to misclassification, specially when based solely on backbone gene homology [23]. Conserved exclusion and partition functions may impair compatibility between IncC and VBR1 plasmids. In this study, we found that pPHDP70 (non conjugative VBR1 plasmid lacking exclusion systems) coexisted with the IncC plasmid pKAZ3 in *E. coli* MC1061 (Table 1). In contrast, we were unable to obtain *E. coli* transconjugants carrying pKAZ3 and pPHDDOG15A, a conjugative VBR1 plasmid encoding exclusion systems. The reasons of this apparent incompatibility are so far unknown, and additional investigation is required to confirm if VBR1 and IncC belong to distinct incompatibility groups.

We found that the VBR1 plasmid pPHDDOG15 can be conjugated from *P. damselae* subsp. *damselae* OG15A to *E. coli* CAG18420 (Table 1), consistent with previous findings [10]. A selected *E. coli* transconjugant for pPHDDOG15A could transfer the plasmid to *E. coli* MG1655 at similar frequency, demonstrating that VBR1 plasmids are replicative and stable in *E. coli* (Table 1). The native *Pdd* strains hosting pPHDDOG2 and pPHDDOG15A maintained these VBR1 plasmids 100% stably over 80 generations, even in the absence of antibiotic selection (data not shown). This stability is likely explained by the presence of a toxin-antitoxin pair conserved in the majority of VBR1 plasmids (Protein ID: KAB1503304.1 and KAB1503305.1 of pPHDDOG2 plasmid). Given the continuous influx of terrestrial and freshwater bacteria into marine environments, there is ample opportunity for horizontal gene transfer across diverse bacterial taxa, potentially bridging terrestrial and aquatic ecosystems. Therefore, it would be expected that VBR1 plasmids might have been transferred to bacterial families beyond *Vibrionaceae*. However, based on currently available GenBank data, VBR1 plasmids appear to be restricted to *Vibrionaceae* species. This suggests that specific host factors may be required for stable maintenance of VBR1 plasmids in natural settings. Nevertheless, the potential for future adaptation and expansion of this replicon type to other bacterial families cannot be excluded.

## Conclusions

This study identifies and characterizes VBR1 as a novel plasmid replicon family widely distributed among *Vibrionaceae*, an ecologically significant bacterial group in marine ecosystems that includes several animal and human pathogens. VBR1 plasmids are highly conserved, predominantly conjugative, and frequently carry AMR determinants, including resistance to compounds used in marine aquaculture and in the treatment of *Vibrio*-associated infections. Our findings underscore the ecological significance of VBR1 plasmids as mobile genetic platforms that drive microbial adaptation in marine ecosystems. By co-localizing AMR genes with phage defense and anti-defense systems, VBR1 plasmids confer a dual advantage: survival under anthropogenic stressors such as antibiotic exposure and resilience against viral predation. This latter feature may have profound ecological implications given the intense phage predation experienced by *Vibrio* populations. The broad distribution of VBR1 across diverse hosts and environments suggests that these plasmids act as hubs for horizontal gene transfer, accelerating the turnover of adaptive traits in vibrios. We propose that VBR1 plasmids contribute to the evolutionary success of *Vibrionaceae* lineages by providing a diverse arsenal of anti-phage defense mechanisms and AMR genes. VBR1 plasmids are stably maintained and transferable across bacterial hosts, yet appear restricted to *Vibrionaceae*, possibly due to host-specific compatibility factors. This work establishes a framework for classifying previously untyped plasmids and introduces a molecular surveillance strategy for high-specificity detection in both laboratory and environmental samples. Continued monitoring of VBR1 plasmids is essential to better understand their ecological dynamics and mitigate their potential impact on public and animal health.

## Supplementary Material

Supplementary_Tables_and_Figures_FINAL

## Data Availability

Data generated or analyzed during this study are included in this published article. Table 2 includes the GenBank accession numbers from all the VBR1 plasmids used in this study. The VBR1 replicon sequence from pPHDDOG2 has been deposited in GenBank under accession number PV872854.
