# Supplementary material for: Replicon family of Vibrionaceae plasmids as a reservoir of antimicrobial and phage resistance genes in marine ecosystems
Source: ISME J. 2025 Dec 10;19(1):wraf274. doi: 10.1093/ismejo/wraf274 (PMC12753305; doi:10.1093/ismejo/wraf274)
Supplement: Supplementary_Tables_and_Figures_FINAL [file supplementary_tables_and_figures_final.pdf]

Supplementary Table S1. Strains and plasmids used in this study.

Strain or plasmid	Description/Relevant characteristics[a]	Reference/Source
**Strains**		
Photobacterium damselae subsp. *piscicida*		
DI21	Isolated from gilthead seabream (*Sparus aurata*), Spain, 1990; pPHDP70	Magariños et al., 1992
DI21-Rif	DI21 spontaneous rifampin resistant strain; Rf^R	Osorio et al., 2006
DS11	Isolated from *Sparus aurata*, Spain, 1995; pPHDP70	Magariños et al., 2000
P. damselae subsp. *damselae*		
OG2	Isolated from seabass (*Dicentrarchus labrax*); pPHDDOG2; Te^R, Cm^R	Vences et al., 2020
OG15A	Isolated from seabass (*Dicentrarchus labrax*); pPHDDOG15A; Cm^R	Vences et al., 2020
Escherichia coli		
DH5α	Cloning strain	Laboratory stock
MG1655-Nal	*E. coli* K12, F-, spontaneous Nl^R mutant	Laboratory stock
MC1061	F^- *araD139* Δ(*ara-leu*)*7696 galE15 galK16* Δ(*lac*)*X74 rpsL hsdR2*($r_K^- m_K^+$) *mcrA mcrB1*	Wertman *et al.*, 1986
CAG18420	MG1655 *lacZU118lacI42*::Tn*10kan*; Kn^R	Singer et al., 1989
**Plasmids**		
pWKS30	Low-copy-number cloning vector, Ap^R	Wang & Kushner, 1991
pKD4	Template for Kn^R gene	Datsenko & Wanner, 2000
pKAZ3	IncC plasmid (*qnrVC1*, *sul1*, *bla*$_{VEB-9}$, *tet*(A)′, *tet*(C), *dfrA1*, *dfrA23*); Ap^R	Flach et al., 2015

[a] Rf^R, rifampin resistance; Kn^R, kanamycin resistance; Cm^R, chloramphenicol resistance; Ap^R, ampicillin resistance; Nl^R, nalidixic acid resistance.

References.

1. Magariños, B., Romalde, J.L., Bandín, I., Fouz, B. & Toranzo, A.E. Phenotypic, antigenic, and molecular characterization of *Pasteurella piscicida* strains isolated from fish. *Appl. Environ. Microbiol.* **58**, 3316–3322 (1992).

2. Osorio, C.R., Juíz-Rio, S. & Lemos, M.L. A siderophore biosynthesis gene cluster from the fish pathogen *Photobacterium damselae* subsp. *piscicida* is structurally and functionally related to the *Yersinia* high-pathogenicity island. *Microbiology* **152**, 3327–3341 (2006).

3. Magariños, B., Toranzo, A.E., Barja, J.L. & Romalde, J.L. Existence of two geographically-linked clonal lineages in the bacterial fish pathogen *Photobacterium damselae* subsp. *piscicida* evidenced by random amplified polymorphic DNA analysis. *Epidemiol. Infect.* **125**, 213–219 (2000).

4. Vences, A. *et al.* Highly transferable pAQU-related plasmids encoding multidrug resistance are widespread in the human and fish pathogen *Photobacterium*

damselae subsp. *damselae* in aquaculture areas in the Black Sea. *Microb. Ecol.* **80**, 507–518 (2020).

5. Wertman, K.F., Wyman, A.R. & Botstein, D. Host/vector interactions which affect the viability of recombinant phage lambda clones. *Gene* **49**, 253–262 (1986).

6. Wang, R.F. & Kushner, S.R. Construction of versatile low-copy-number vectors for cloning, sequencing and gene expression in *Escherichia coli*. *Gene* **100**, 195–199 (1991).

7. Datsenko, K.A. & Wanner, B.L. One-step inactivation of chromosomal genes in *Escherichia coli* K-12 using PCR products. *Proc. Natl Acad. Sci. USA* **97**, 6640–6645 (2000).

8. Singer, M. *et al.* A collection of strains containing genetically linked alternating antibiotic resistance elements for genetic mapping of *Escherichia coli*. *Microbiol. Rev.* **53**, 1–24 (1989).

9. Flach, C.F. *et al.* Isolation of novel IncA/C and IncN fluoroquinolone resistance plasmids from an antibiotic-polluted lake. *J. Antimicrob. Chemother.* **70**, 2709–2717 (2015).

Supplementary Table S2. Oligonucleotides used in this study.

Oligonucleotides	Sequence (5' → 3')	Amplicon size (bp)
**Determination of the replication origin of pPHDP70**		
kanR_pKD4_BamHI_5	gcGGATCCtagaaagccagtccgcagaa	1193
kanR_pKD4_BamHI_3	gcGGATCCgaagcccaacctttcataga	
Seq2_BamHI_5	gcGGATCCgcctgaacctgacgacattt	6808
Seq2_BamHI_3	gcGGATCCcgagcaggggccaattatta	
Seq1_ BamHI_ F	gcGGATCCccttcaagaagaatcacagc	2843
Seq1_ PstI	gcCTGCAGggtttgttactcataggatg	
Seq3_BamHI	gcGGATCCgcactaggaactataagtgc	578
Seq3_BamHI	gcGGATCCcctaaccttcaagtaatcca	
**Surveillance of VBR1 plasmids**		
Seq3_R	gcactaggaactataagtgc	578
Seq3_F	cctaaccttcaagtaatcca	
Seq1_F	ctaaacagcatctgagtcgcc	505
Seq1_R	tcggtctttgatgctggtgg	

Supplementary Figures.

Figure S1. Size distribution of VBR1 plasmids listed in Table 1 and 2 (n=114), all expected to be fully assembled. Plasmid sizes range from 70 kb to 270 kb.

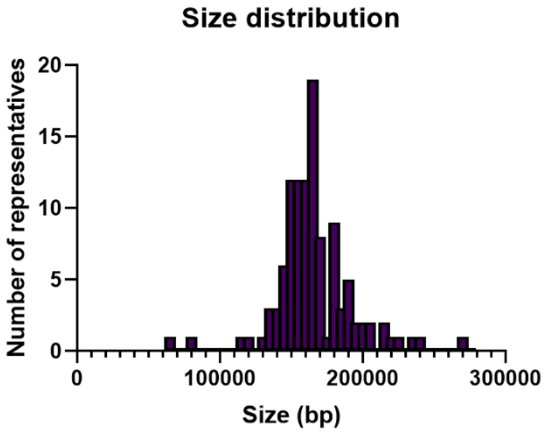

Figure S2. Extended heat map showing the presence/absence profiles of antibiotic resistance genes across 114 strains belonging to the *Vibrionaceae* family.

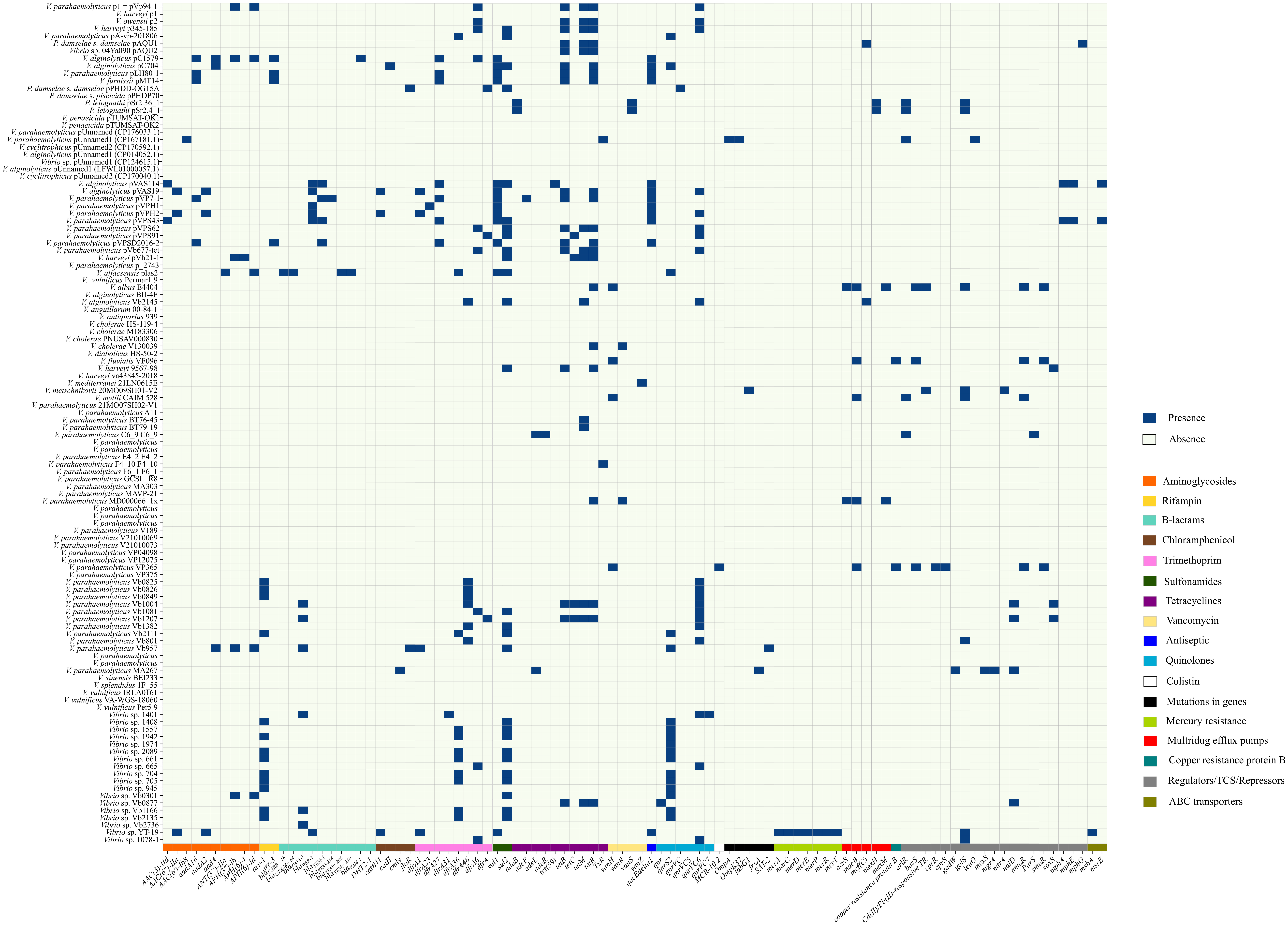

Figure S3. PCR-based detection of VBR1-family plasmids in environmental samples. Numbers in parentheses indicate the number of positive samples relative to the total number of samples analyzed

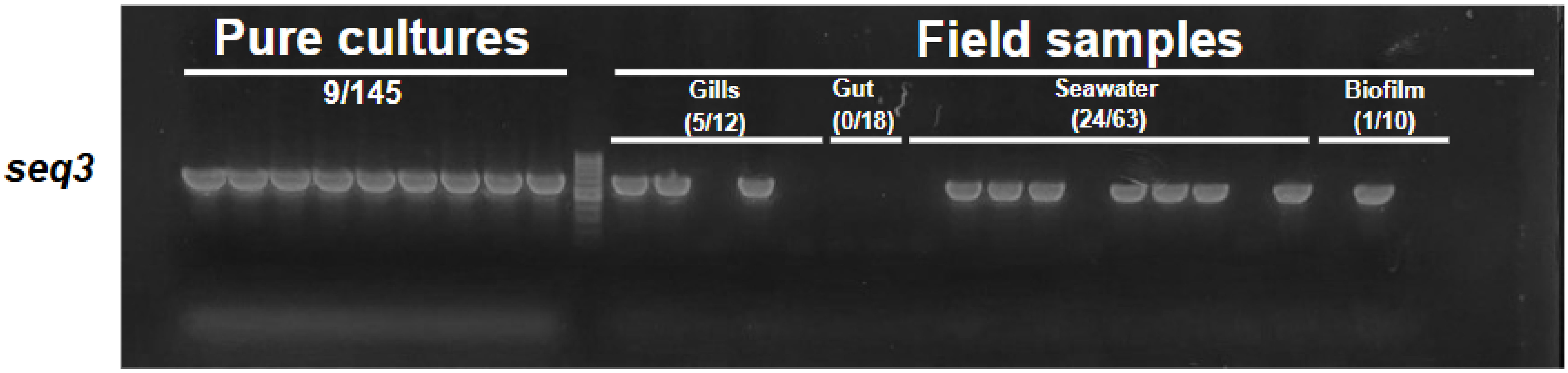

Figure S4. Extended heat map showing the presence/absence profiles of phage defense and anti defense genes across 158 strains belonging to the *Vibrionaceae* family.

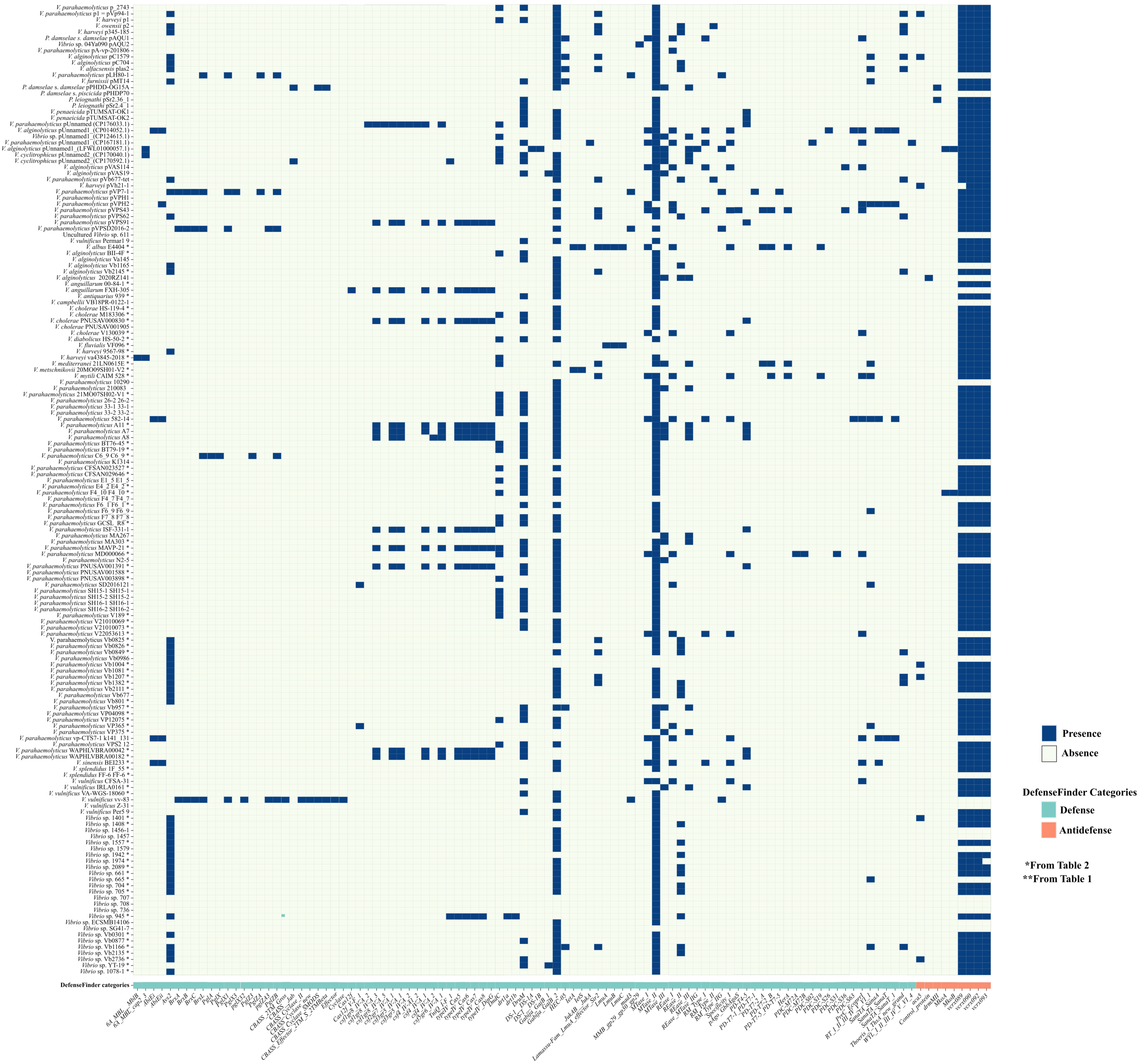

Figure S5. Phylogenetic trees of (A) Vrp1A and (B) Vrp1B amino acid sequences; and of (C) *oriV* nucleotide sequences of selected VBR1 plasmids.

A

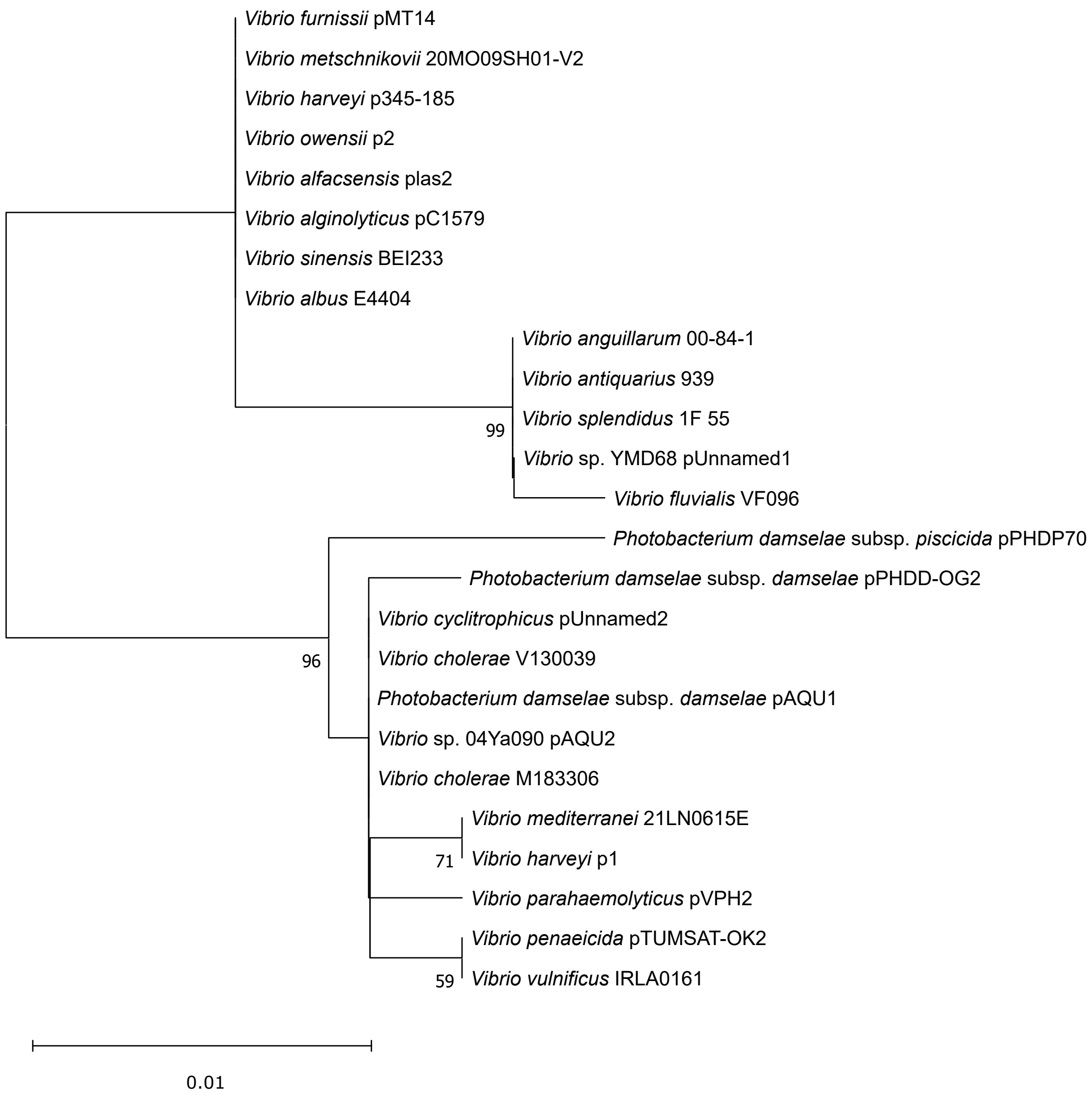

B

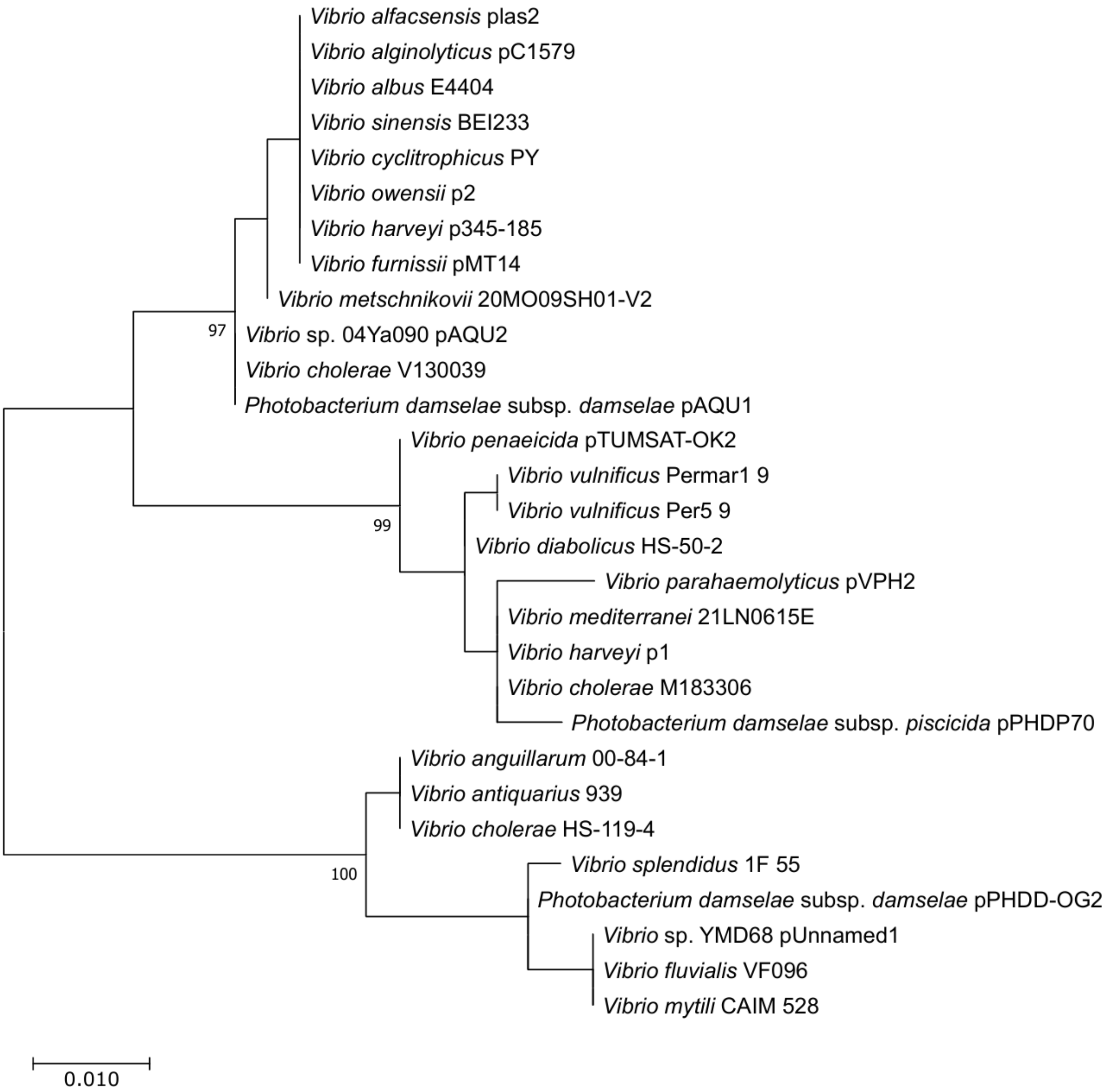
Vibrio alfacsensis plas2
Vibrio alginolyticus pC1579
Vibrio albus E4404
Vibrio sinensis BEI233
Vibrio cyclitrophicus PY
Vibrio owensii p2
Vibrio harveyi p345-185
Vibrio furnissii pMT14
Vibrio metschnikovii 20MO09SH01-V2
97
Vibrio sp. 04Ya090 pAQU2
Vibrio cholerae V130039
Photobacterium damselae subsp. damselae pAQU1
Vibrio penaeicida pTUMSAT-OK2
Vibrio vulnificus Permar1 9
Vibrio vulnificus Per5 9
99
Vibrio diabolicus HS-50-2
Vibrio parahaemolyticus pVPH2
Vibrio mediterranei 21LN0615E
Vibrio harveyi p1
Vibrio cholerae M183306
Photobacterium damselae subsp. piscicida pPHDP70
Vibrio anguillarum 00-84-1
Vibrio antiquarius 939
Vibrio cholerae HS-119-4
Vibrio splendidus 1F 55
100
Photobacterium damselae subsp. damselae pPHDD-OG2
Vibrio sp. YMD68 pUnnamed1
Vibrio fluvialis VF096
Vibrio mytili CAIM 528
0.010

C

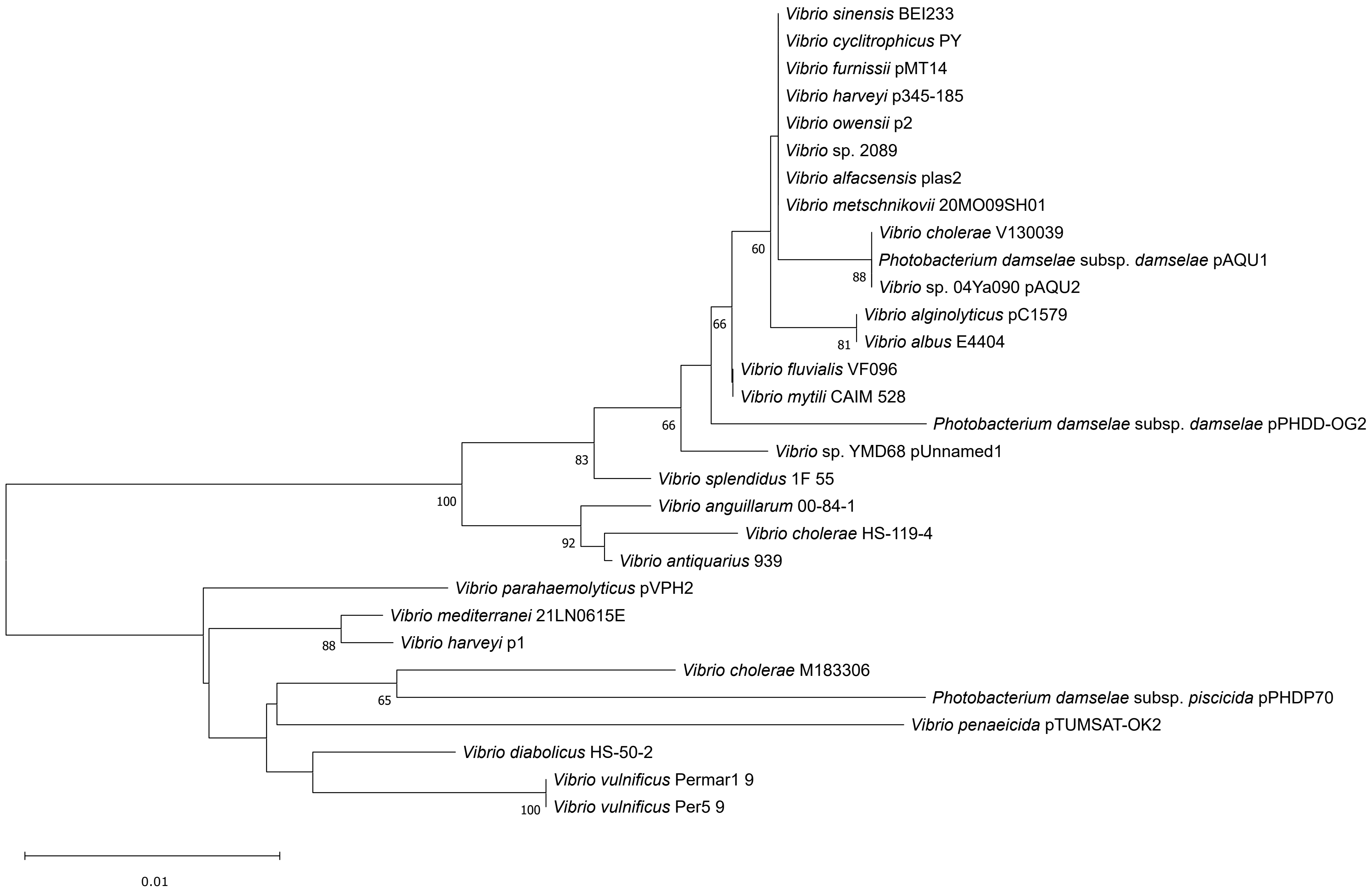
Vibrio sinensis BEI233
Vibrio cyclitrophicus PY
Vibrio furnissii pMT14
Vibrio harveyi p345-185
Vibrio owensii p2
Vibrio sp. 2089
Vibrio alfacsensis plas2
Vibrio metschnikovii 20MO09SH01
Vibrio cholerae V130039
Photobacterium damselae subsp. damselae pAQU1
Vibrio sp. 04Ya090 pAQU2
Vibrio alginolyticus pC1579
Vibrio albus E4404
Vibrio fluvialis VF096
Vibrio mytili CAIM 528
Photobacterium damselae subsp. damselae pPHDD-OG2
Vibrio sp. YMD68 pUnnamed1
Vibrio splendidus 1F 55
Vibrio anguillarum 00-84-1
Vibrio cholerae HS-119-4
Vibrio antiquarius 939
Vibrio parahaemolyticus pVPH2
Vibrio mediterranei 21LN0615E
Vibrio harveyi p1
Vibrio cholerae M183306
Photobacterium damselae subsp. piscicida pPHDP70
Vibrio penaeicida pTUMSAT-OK2
Vibrio diabolicus HS-50-2
Vibrio vulnificus Permar1 9
Vibrio vulnificus Per5 9
60
88
66
81
66
83
100
92
88
65
100
0.01
